# The short-term effect of a modified comprehensive geriatric assessment and regularly case conferencing on neuropsychiatric symptoms in nursing homes: a cluster randomized trial

**DOI:** 10.1186/s12877-022-02976-x

**Published:** 2022-04-11

**Authors:** Geir-Tore Stensvik, Anne-Sofie Helvik, Gørill Haugan, Aslak Steinsbekk, Øyvind Salvesen, Sigrid Nakrem

**Affiliations:** 1 Department of Public Health and Nursing, Faculty of Medicine and Health Sciences, Norwegian University of Science and Technology (NTNU), Trondheim, Norway; 2 Norwegian National Advisory Unit On Ageing and Health, Vestfold Hospital Trust, Tønsberg, Norway; 3 Faculty of Nursing and Health Sciences, Nord University, Levanger, Norway; 4 Norwegian Centre for E-health Research, Tromsø, Norway

**Keywords:** Behavioural symptoms, Case conferencing, Cluster RCT, Cognitive impairment, Comprehensive geriatric assessment, Dementia; Long term facilities, Neuropsychiatric symptoms, Nursing Home

## Abstract

**Aims:**

To investigate the short-term effect of implementing a modified comprehensive geriatric assessment and regularly case conferencing in nursing homes on neuropsychiatric symptoms.

**Background:**

Neuropsychiatric symptoms are common and may persist over time in nursing home residents. Evidence of effective interventions is scarce.

**Design:**

A parallel cluster-randomised controlled trial.

**Methods:**

The intervention was monthly standardised case conferencing in combination with a modified comprehensive geriatric assessment. The control group received care as usual.

Main outcome measure.

The total score on the short version of the Neuropsychiatric Inventory (NPI-Q, 12-items).

**Results:**

A total of 309 residents at 34 long-term care wards in 17 nursing homes (unit of randomisation) were included. The intervention care units conducted on average two case conference-meetings (range 1–3), discussing a mean of 4.8 (range 1–8) residents. After 3 months, there were no difference of NPI-Q total score between the intervention (-0.4) and the control group (0.5) (estimated mean difference = -1.0, 95% CI -2.4 to 0.5, *p* = 0.19). There was a difference in favour of the intervention group on one of the secondary outcome measures, the apathy symptoms (-0.5 95% CI: -0.9 to -0.1, *p* = 0.03).

**Conclusion:**

In this study there were no short-term effect of case conferencing and modified comprehensive geriatric assessments after three months on the total score on neuropsychiatric symptoms. The intervention group had less apathy at 3 months follow-up compared to those receiving care as usual. The findings suggest that a more comprehensive intervention is needed to improve the total Neuropsychiatric symptoms burden and complex symptoms.

**Trial registration:**

Due to delays in the organisation, the study was registered after study start, i.e. retrospectively in Clinicaltrials.gov # NCT02790372 at https://clinicaltrials.gov/; Date of clinical trial registration: 03/06/2016.

## Background

Nursing home (NH) residents are often frail older adults with complex needs due to several concurrent chronic conditions, including dementia [1]. However, the diversity of residents’ needs, ranging from social care needs to palliative care needs, adds to the complexity of NH care [2]. The typical NH residents in Norway, are 85 years on average and approximately 80% have dementia [3].

About 90% of those living with dementia develop at least one neuropsychiatric symptom (NPS) such as agitation, aggression, apathy and depression [4] during the course of their disease [5, 6]. Several reports have defined specific subsyndromes, aggregated into groups of NPS consisting of agitation (agitation/aggression, disinhibition and irritability), affective (depression and anxiety), psychosis (delusions and hallucination), and apathy [6]. Many NPS persist over time [6]. The agitation sub-syndrome and apathy are among the very prevalent and persistent NPS [7]. The severity of affective symptoms, apathy and psychosis can remain relatively stable over time [8].

The aetiology of NPS is mostly unknown, but factors such as neuropathological changes in the brain, unmet psychosocial needs and physical health problems are of importance [9, 10]. Infections, pain and dehydration exemplify common health problems associated with inclining NPS [11–13]; these health problems possibly relate to nursing care quality and are therefore modifiable [14, 15]. For people with dementia, non-pharmacological interventions, i.e. psychosocial interventions should be used as the first-line treatment to manage the NPS [16]. Furthermore, to modify NPS, person-centred care and individualised interventions are recommended [16, 17] along with an evaluation of all possible root causes of NPS [18, 19].

### Comprehensive geriatric assessment and case conferencing

The term ‘geriatric assessment’ most commonly refers to a clinician’s (primary care clinician or a geriatrician) evaluation of an older adult’s health conditions [20]. However, ‘geriatric assessment’ is also used when referring to a more intensive multidisciplinary program, known as a Comprehensive Geriatric Assessment (CGA) [20] which is an established method in clinical practice to assess older adult’s health [21]. CGA is performed at varying levels of intensity in different settings; accordingly, its content may vary with the healthcare setting [22]. To identify health problems that can be treated for better health outcomes, CGA starts with a systematic evaluation of the adult [20, 21], followed by an individual care plan that explicitly states the individual’s goals of care, who is responsible for achieving these goals, and a timeline for review of progress. Some studies show that the use of CGA contribute to less hospitalizations, higher care quality and lower mortality [20].

Case Conferencing represents one way to implement CGA and follow-up individual care plans [23]. In the following text, the abbreviation CC refers to the case-conferencing working process, while the term CC-meeting refers to the arranged meetings for case-conferencing. The CC-approach promotes a person-centred care perspective since it involves individualised interventions aiming at assessing unmet needs and health problems, thereby the potential to modifying NPS [24]. CC is shown to facilitate communication and coordination between nursing home staff [25]. Regular CC-meetings enable the NH staff to communicate in a structured, systematic and goal-oriented way, and thereby establish a common thought-through understanding of each case [26]. This approach ensure that the resident’s individual needs can be identified and individualised care interventions developed [26].

Even so, research investigating the effect of CC regarding NPS in dementia care is scarce [24]. A systematic review from 2012 reported positive effects of CC on NPS in four out of seven studies [24]. However, none of included studies used CGA as a part of the intervention nor did they use the Neuropsychiatric Inventory-Questionnaire [27] but used other measurements. A recent cluster-randomised controlled trial (c-RCT) study from 2016 reporting short term effects found that a multicomponent intervention including similar approach and comprehensive assessment significantly reduced agitation in NH residents at 8 and 12 weeks [28]. Summarized, large high-quality studies on the effect of CC combined with CGA on NPS in NH are needed [24].

The primary aim of the present study was to investigate the short-term effects of implementing modified CGA (m-CGA) and regularly CC compared to standard practice on total neuropsychiatric symptoms load in nursing home residents. The secondary aim was to investigate the effect on important NPS including the subsyndromes agitation (agitation/aggression, disinhibition and irritability), affective (depression and anxiety), psychosis (delusions and hallucination) and apathy (one item).

## Methods

### Study design

This was a parallel group c-RCT. The cluster design was chosen to avoid contamination as the intervention was targeted towards the entire staff and potentially all residents in the nursing homes. The study was conducted between April 2015 and May 2016. The CONSORT checklists with extension for cluster-randomised trials and non-pharmacologic trials were used to guide the reporting [29, 30]. There were no changes to the methods after trial commencement. This paper reports the outcome after three months on primary outcome, total NPS, and secondary outcomes on important NPS subsyndromes and apathy. The trial was registered in ClinicalTrial.gov 03/06/2016 (NCT 02,790,372) and approved by the Regional Ethical committee for Medical Research in Western Norway (2014/1642).

### Setting

In Norway, the municipalities are responsible for the NH service which are mostly public, financed by taxes and up to 85% of the residents’ retirement income [31]. These NH are typically organised with a director for the whole NH, a varying number of physically separated wards with ward managers, and a number of care units within each ward. Usually, the ward managers are registered nurses (RN). A care unit typically has six to eight beds with one RN and one (at night) to four (during daytime) Licensed Practical Nurses (LPN) along with varying numbers of unskilled nursing assistants. A general practitioner has the medical responsibility and visits the nursing home 1–2 times per week. In Norway there are no established guidelines for admitting persons with dementia and NPS to a special care unit or to geriatric psychiatry. Residents with dementia can be admitted to a special care unit if the nursing home resident demonstrates a challenging behavior for the staff or other residents and if there are available beds. In many cases the nursing home staff will consult geriatric psychiatry ahead of admission.

### Participants

Inclusion criterion for the NH was acceptance to partake in the trial. Only regular care units were invited. Special dementia care units with enhanced staffing were excluded. For each unit, at least one RN with at least 3 years work experience and employed in minimum 75% position during the study period was in charge of conducting the intervention.

Inclusion criteria for the residents were: (1) long-term stay, (2) residential time ≥ 60 days, (3) life expectancy ≥ 6 months (evaluated by the RN), and (4) informed consent to participate signed by the resident. In cases where the resident was unable to provide informed consent, the RN informed the next-of kin about the study and asked for consent on behalf of the resident.

### Procedure

The recruitment was done in three steps: (1) the NH, (2) the RN in the care units and (3) the residents. NH managers received an e-mail informing them about the study and inviting the NH to participate. The NH managers who accepted to participate recruited the RN based on the inclusion criteria. Participating RN recruited the residents based on the specified inclusion criteria. Before study start, all participating RNs were trained in using the assessment tools used for measuring outcome (see below). These RNs also arranged CC-meetings and were not blinded after allocation to intervention. All NH received payment to compensate for time used on data collection. NH allocated to intervention got additional training in how to conduct the intervention. The participating staff members (RN, LPN and assistants) received the training and performed the m-CGA and CC-meetings during their ordinary working hours. The intervention NH received payment to compensate for training, The NH did not receive additional resources during the study period, rendering their work processes comparable to normal operation. The NH were given facilitation on when to perform the CC-meeting through the study period, but they were in charge on how they would implement them.

### Intervention

The intervention comprised a monthly m-CGA of the residents included in the study using validated instruments assessing common physical and psychological health problems associated with NPS, followed by a monthly structured CC-meeting discussing and developing an individual care plan. The participating RN performed m-CGA to facilitate clinical decision-making, and then arranged a CC-meeting with the care team in the NH (Table 1). Two experienced university lecturers in nursing, including the first author, provided a standardized four-hour intervention training course for the NH management and RN separately for each NH (Table 1). The number of participating RN per unit in the training sessions and time from training to inclusion were recorded in a protocol.

**Table 1:** Overview of the intervention content, training, and support prior to the study

Element of intervention	Content	Training and support
Modified Comprehensive Geriatric Assessment (m-CGA) tool- box	The Physical Self-Maintenance Scale (PSMS) Cornell Scale for Depression in Dementia (CSDD) Clinical Dementia Rating (CDR) scale Neuropsychiatric Inventory-Questionnaire (NPI-Q, 12-ITEMS) The quality of life in late-stage dementia (QUALID) The Brief Agitation Rating Scale (BARS) a subscale of the Cohen-Mansfield Agitation Inventory (CMAI) The 24-h registration of behaviour form	A 30 min’ lecture on how to use assessments for case conferencing Written educational material
Case conference meeting	Four structured steps:	
	1) Evaluate effects of previous nursing interventions based on updated assessment 2) Create a common understanding of the problem or area for improvement 3) Determination of concrete and realistic goal of care 4) Discuss, decide and define nursing interventions and appropriate method for evaluation	A 45 min’ lecture on symptoms, causes and explanations of neuropsychiatric symptoms A 45 min’ lecture on why and how to perform a case conference A 30 min’ practical training session in performing a case conference (using a resident case from the actual nursing home as example) Written educational material and a manual for structuring the case conference
Documentation and reporting (using Electronic Patient Record)	Care plan should be updated after each case conference by updating the electronic record (nursing module)	A 45 min’ lecture on the nursing care process including demonstration of resident example
Additional assessments (when the resident’s symptoms/needs, or situation requires it)	The brief agitation rating scale (BARS), 24-h registration of behaviour form	A 30 min’ lecture on how to use assessments for case conferencing Written educational material

### Case conference meetings

A RN in each of the care units who participated in the intervention-training course arranged the CC-meetings together with at least two LPN in line with the structure and recommendations shown in Table 1. During the CC-meetings, the participants had specific roles, a chairperson or responsible for the minutes and care plan documentation, in accordance with detailed written information provided by the researchers. The RN arranged at least one monthly CC-meeting for all included residents. About 20 min were scheduled per resident. To avoid meetings exceeding 90 min, care units with more than six residents arranged two monthly CC-meetings. Further details about the CC-meetings are published previously [32].

### Modified Comprehensive Geriatric Assessment (m-CGA)

The RN in each unit assessed the NH residents by a set of recommended geriatric assessment instruments (see Table 1 and procedures above). The results of the assessment were then used for support in the clinical decision process during the CC-meetings.

- The Physical Self Maintenance Scale (PSMS) [33] assessed performance of activities of daily living. This scale consists of six items scaled from total independence [1] to total dependence [5], with a total score ranging from 6–30. Higher score indicates greater dependency.
- The Cornell Scale for Depression in Dementia (CSDD) [34], Norwegian version [35], assessed depression symptoms. The score ranges between 0 and 38; higher scores indicate more depressive symptoms. The CSDD has recently been validated in Norwegian NH residents and the psychometric properties are acceptable [36].
- The Clinical Dementia Rating (CDR) scale assessed severity of dementia. The CDR covers six domains (memory, orientation, judgment and problem solving, community affairs, home and hobbies, and personal care). Each domain has five response categories (0, 0.5, 1, 2, 3) [37, 38]. The CDR standard global score is calculated by means of an algorithm giving priority to memory (https://www.alz.washington.edu/cdrnacc.html).
- Quality of Life in Late-Stage Dementia (QUALID) scale [39], Norwegian version [40]. For each resident, the frequency of 11 observable behaviours during the previous week was registered (total score range 11–55). A higher score indicates poorer QoL.
- The Brief agitation rating scale (BARS) [41] is a subscale of the Cohen-Mansfield Agitation Inventory (CMAI) [42]. If any NPI-Q aggression/agitation items score was ≥ 1, the BARS was included in the monthly m-CGA. The Norwegian version of BARS [43] consists of 10 items: hitting, pushing, grabbing, pacing, restlessness, repetitive sentences, and repetitive mannerisms, complaining and making strange noises. The frequencies of these symptoms are rated from 1 (never) to 7 (several times per hour), reporting the frequency of the agitated behaviour during the preceding 2 weeks retrospectively. The sum score ranges from 10 to 70, with a higher score indicating more agitation [41].
- The 24-h registration of behaviour form includes a table showing hours in the horizontal rows and days in the columns. By using different colour codes for the various observed behaviours, this form visualizes when the observed behaviour occurs. The observation period could be a week or more. This symptom profile identifies and helps to understand environmental triggers of behaviour. Furthermore, this form portrays the frequency of behavioural events throughout the day (https://www.aldringoghelse.no/skalaer-og-tester/#dognregistreringsskjema-registreringsskjema-for-atferd). It was utilized as an additional measure in the m-CGA to help the staff getting a clearer picture of NPS.

## Measures

All participants' age, gender and clinical background (PSMS, CSDD and CDR were assessed at baseline (Table 2).

## Outcomes

This paper reports on the outcomes measured with the short version Neuropsychiatric Inventory-Questionnaire (NPI-Q, 12-items) [44]. The NPI-Q is adapted from the NPI, a validated informant-based interview that assesses NPS during the previous month [27]. The NPI-Q includes the following 12 NPS: delusion, hallucination, agitation/aggression, depression/dysphoria, anxiety, apathy/indifference, irritability/lability, euphoria, disinhibition, aberrant motor behaviour, night-time behaviour, and appetite and eating disorders, one item each. These symptoms are registered as present or not. If present, the severity of the symptom is scaled from 1 (Mild), 2 (Moderate) to 3 (Severe), which gives a range in total score from 1 to 36 (higher score indicates more severe symptoms). The NPI-Q was assessed at baseline and after three months in both groups.

### Primary outcome

The primary outcome was the change from baseline to three months in total score of NPI-Q 12 items.

### Secondary outcomes

The secondary outcomes consisted of changes from baseline to three months in the score of the three sub-syndrome sum-scores of the NPI-Q 12 items: 1) agitation constructed by three items (agitation/aggression, irritability, and disinhibition giving a score range of 0–9), 2) affective symptoms (depression and anxiety, score range 0–6), 3) psychosis (delusions and hallucination, score range 0–6) and 4) apathy symptoms (a single item, score range 0–3) [45].

## Intervention compliance

The RN chairing the CC-meeting recorded in a logbook the date for the meeting, who participated, and the initials of the resident in focus. The logbook was collected at the end of the trial. Based on these logs, the number of CC-meetings per resident and care unit as well as number of residents per CC-meeting and the attending staff were calculated.

## Sample size

The sample size was calculated to detect an effect size of 0.4 (small to moderate) on NPI-Q [46]. Based on previous research, we assumed an intra-cluster correlation coefficient (ICC) within the NH of *p* = 0.04 [46]. A power of 80% with a significance level of 5% required 380 residents in 10 intervention clusters and 10 control clusters, with on average 19 residents in each cluster. No information about the actual cluster-sizes were available when performing the power calculation, thus an equal cluster-size was assumed. The calculations were performed with IBM SPSS Sample Power 3.0 (IBM Corp., Armonk, N.Y., USA).

## Randomisation and allocation

The NH was defined as a single cluster and was randomised. We assumed that the NH size could influence on how the intervention was delivered; therefore, the randomisation included stratification for NH size into three blocks: small (9–25 residents; 4 NH), medium (26–59 residents; 11 NH), and large (> 60 residents; 2 NH). The trial service at Norwegian University of Science and Technology conducted a computer-generated randomisation and kept the allocation concealed until the baseline data were completed. Information about allocation was then given to the first author (GTS) who informed the NH managers about the allocation by e-mail.

## Statistical methods

A statistician with no knowledge about the intervention contributed to the analyses. There was some skewness in most variables, however, overall, the baseline characteristics and outcome variables seemed approximately normally distributed. The baseline characteristics of the groups were compared using independent sample t-test for continuous variables and a Chi-squared test for categorical variables. The outcomes were analysed according to intention-to-treat (ITT). Mixed linear models assessed the effects of the intervention, with change in scores from baseline to 3 months follow-up as the dependent outcome variable and treatment specified as a fixed effect. To allow for a potential cluster effect on change scores, the ID of the nursing homes was included as a random effect and the intra-cluster correlation coefficient (ICC) on change scores is reported [47]. Statistical calculations were performed using the software R (The R Project for Statistical Computing), version 2.131 and the IBM SPSS Statistics for Windows, version 22 (IBM Corp., Armonk, N.Y., USA).

In addition, a per-protocol analysis was conducted. The criterion for per-protocol was that the care units completed at least 50% of the CC-meetings with at least one RN and two LPNs present.

## Results

**Fig. 1:**
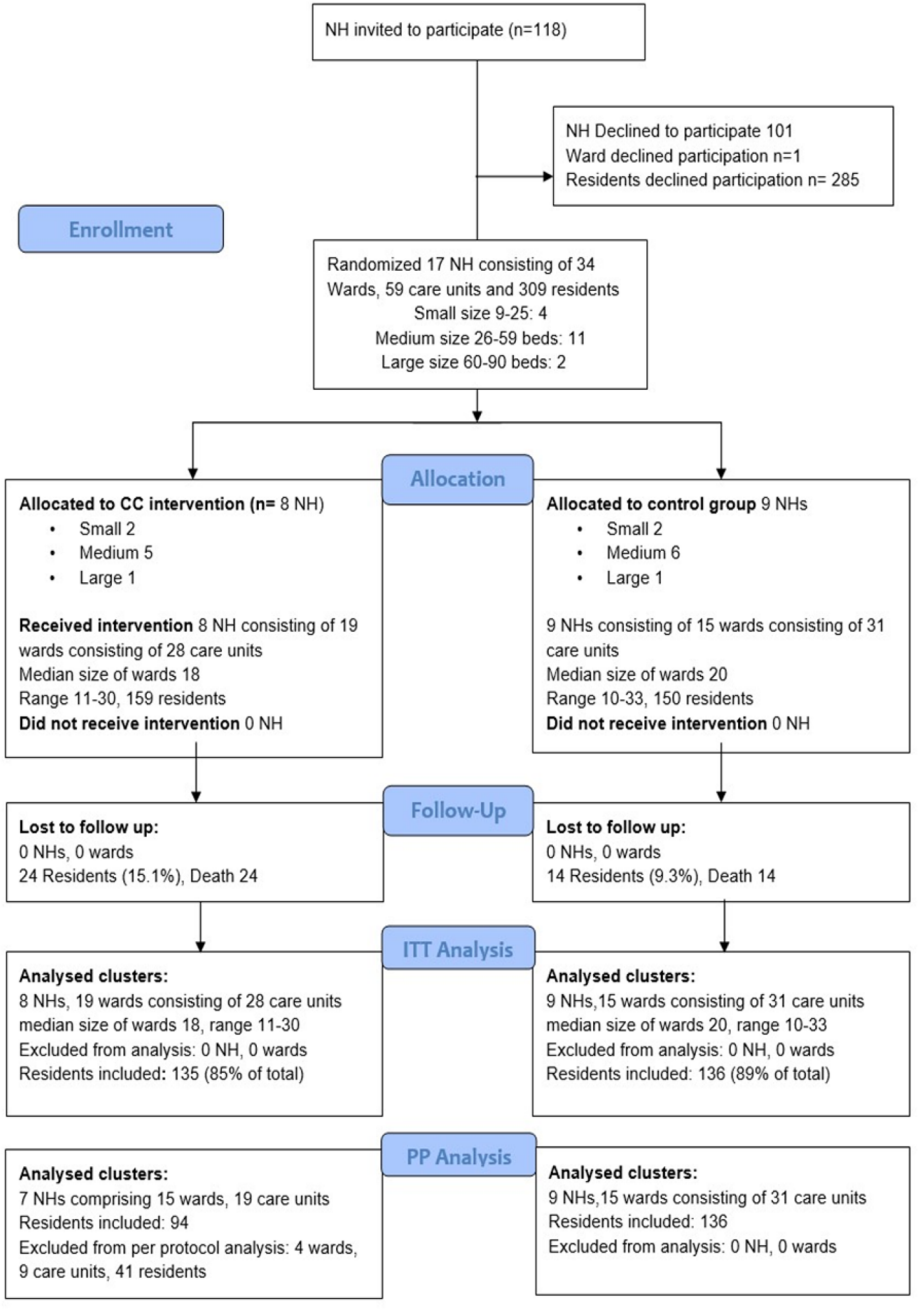
Flow chart

A total of 118 NH was approached, out of which 17 NH participated (Fig. 1). The participating NH had totally 35 wards, 61 care units and 594 residents. Of these, 309 residents (55.6% of those eligible) in 59 care units organised into 34 wards participated.

Eight NH (159 residents, 19 wards organised in 28 care units) were randomised to the intervention group and nine NH (150 residents in 15 wards organised in 29 care units) to the control group. The groups were similar at baseline (Table 2) with 68% being female, and the mean number of residents per care unit in the interventions group was 5.7 (range 1–13) and 5.2 (range 1 to 11) in the control group.

**Table 2:** Resident and nursing home characteristics at baseline

Characteristics	Unit	Total	INT	CTR	P value
*Patients characteristics*		*(n* = *309)*	*(n* = *159)*	*(n* = *150)*	
Women	N (%)	210 (68)	109 (69)	101(67)	0.82 ^a^
Age years	Mean ± SD	85.4 ± 8.1	85.5 ± 8.3	85.2 ± 8.0	0.80^b^
PSMS	Mean ± SD	17.2 ± 5.3	17.5 ± 5.2	16.9 ± 5.5	0.36^b^
CSDD	Mean ± SD	4.8 ± 5.1	5.0 ± 5.2	4.6 ± 5.0	0.51^b^
CDR	Mean ± SD	14.8 ± 5.6	14.3 ± 5.0	15.1 ± 6.0	0.28^b^
Stay in NH, months	Mean ± SD	29.9 ± 29.0	31.7 ± 31.6	27.9 ± 25.9	0.25^b^
*NH characteristics*		*(N* = *17)*	*(N* = *8)*	*(N* = *9)*	
Number of wards per NH	Mean ± SD	2.0 ± 0.9	2.3 ± 1.2	1.7 ± 0.5	0.12^b^
Number of care units per NH	Mean ± SD	3.5 ± 1.7	3.5 ± 1.5	3.3 ± 1.8	0.80^b^
Number of residents per NH	Mean ± SD	18.2 ± 9.4	19.9 ± 9.6	16.7 ± 9.6	0.50^b^
Number of residents per care unit	Mean ± SD	5.4 ± 2.8	5.7 ± 3.3	5.2 ± 2.2	0.50^b^

*INT* Intervention group, *CTR* Control group, *CDR* Clinical Dementia Rating scale, *NH* Nursing home, *PSMS* Physical Self Maintenance Scale, *SD* Standard Deviation

^a^*p* value for χ2 test; ^b^*p* value for independent t-test

### Drop-outs

Compared to those alive after three months, the 38 residents who died had a significant higher baseline score on PSMS and CDR. The 271 remaining residents had no missing data.

### Implementation of the intervention

In total 36 RNs from the 28 intervention care units participated in the CC training course. The time between baseline assessment and intervention initiation varied between 5 to 9 days. During the three months intervention period, the intervention care units conducted on average two CC-meetings (range 1–3), with a mean of 4.8 (range 1–8) residents discussed at each CC-meeting. On average, each resident was discussed on 2.1 (range 1- 3) CC-meetings. Usually, one RN (range 1–2) and two LPNs (range 1–4) were present at the CC-meetings. Nineteen (68%) of the 28 care units performed at least two CC-meetings with one RN and two LPNs present. The nursing staff agreed upon the most urgent health problems or unmet needs which they wanted to target without use of pharmacological intervention (Table 1).

### Between-group differences

The ITT analyses revealed no difference of change in NPI-Q 12 items total score between the intervention (-0.4) and the control group (0.5) (estimated mean difference = -1.0, 95% CI -2.4 to 0.5, *p* = 0.19) (Table 3).

**Table 3:** Intention to treat analyses of the between and within group changes from baseline to three months

Group (Number of residents with symptoms)		Baseline INT *n* = 159 CTR *n* = 150	3 months INT *n* = 135 CTRL *n* = 136	Between groups at 3 months		Within groups (3 months–baseline)		ICC
		Mean ± SD	Mean ± SD	Est. diff. (95% CI)	p-value	Change (95%CI)	p-value	
Total NPI-Q	INT (129)	4.5 ± 5.2	3.9 ± 3.7	-1.0 (-2.4, 0.5)	0.19	-0.4 (-1.4, 0.6)	0.42	0.07
(Range 0–36)	CTRL (121)	4.9 ± 5.4	5.4 ± 6.0			0.5 (-0.5,1.6)	0.30	
Agitation	INT (76)	1.4 ± 1.9	1.5 ± 2.0	-0.2 (-0.8, 0.4)	*0.54*	0.1 (-0.3,0.5)	0.64	0.03
(Range 0–9)	CTRL (83)	1.7 ± 2.1	2.0 ± 2.5			0.3 (-0.1,0.7)	0.18	
Affective	INT (60)	0.7 ± 1.1	0.6 ± 1.0	0.05 (-0.2, 0.3)	*0.67*	-0.1 (-0,3, 0.1)	0.40	0.00
(Range 0–6)	CTRL (62)	1.0 ± 1.4	0.8 ± 1.4			-0.1 (-0.3, 0.0)	0.15	
Psychosis	INT (68)	0.8 ± 1.2	0.8 ± 1.2	-0.25 (-0.5, 0.1)	*0.11*	-0.07 (-0.3,0.2)	0.55	0.04
(Range 0–6)	CTRL (63)	0.8 ± 1.2	0.9 ± 1.3			0.18 (-0.0,0.4)	0.10	
Apathy	INT (43)	0.7 ± 1.1	0.5 ± 0.8	-0.5 (-0.9, -0.05) *	*0.03*	-0.2 (-0.5,0.1)	0.24	0.10
(Range 0–3)	CTRL (37)	0.6 ± 1.1	0.9 ± 1.3			0.3 (-0.01,0.6)	0.06	

The between group estimated mean differences and the within-group group differences have been calculated using mixed linear model

*INT* Intervention group, *CTR* Control group, *CI* Confidence Interval, *NPI-Q*, 12-items: Neuropsychiatric inventory, 12-items, *ICC* model based Intra Class Correlation on change score. Coefficient, *SD* standard Deviation

^*^p-value ≤ 0.05

For the secondary outcomes, the NPI-sub-syndromes agitation, affection, and psychosis, there was no effects of the intervention (Table 3). For the single item on apathy, there was a difference in favour of the intervention group (estimated mean difference = -0.5, 95% CI -0.9 to -0.05, *p* = 0.03). The per-protocol analysis gave the similar results as the intention to treat analysis and is thus not reported.

### Within-group changes

There were no within-group changes for any of the outcomes from baseline to 3 months (Table 3).

## Discussion

The present c-RCT study aimed to investigate if m-CGA in combination with the CC-intervention for 3 months could reduce NPS in general. There were no short-term effects of the m-CGA and CC-intervention on changes in neuropsychiatric total symptom score (NPI-Q 12 items), but there was an effect on the secondary outcome apathy.

The NPS total score was chosen as the primary outcome since the resident’s limitations, needs and difficulties probably contribute to NPS [11–13, 15]. However, no between-group or within-group effect of the intervention was found on the primary outcome. The nursing intervention on a specific NPS such as agitation will vary from interventions of other types of symptoms. This was supported in another c-RCT aiming to reduce NPS in Norwegian NH residents that was ongoing simultaneously with our study [28]. This study used a multi-component and multi-disciplinary intervention program for three months, and the primary aim for their intervention was to create a mutual understanding of NPS and to tailor a detailed treatment including both pharmacological and non-pharmacological actions to reduce agitation [28]. The secondary outcomes concerning the total NPS score, apathy or any sub-syndrome in this study was not significant [28].

High age, frailty and infirmities characterize the Norwegian NH population [48]. This was also the case in this study, making the findings generalizable as all residents in the participating care units were included regardless of level and type of NPS. This is a likely an explanation why their NPS load at baseline was quite mild, with a mean NPI-Q score of 4.8 (range 0–36). The effect of the CC-intervention, if implemented in residents with higher intensity or directed toward a specific NPS like aggression remains unknown. Further studies focusing on NPS can thus be recommended to have an inclusion criteria for NPS load to ensure that the residents in most need of interventions targeting NPS symptoms are included. It may also indicate that interventions in studies similar to our study should be more specific and targeted toward specific symptoms. All residents in the intervention NH were included and no specific type of NPS were prioritised, rather the care plans comprised a multimodal and holistic set of individualised nursing interventions.

The present study showed a between-group difference after three months in favour of the intervention group on apathy. We do not have a firm explanation for why a between-group difference was found for apathy and not for affective or psychotic field of NPS. However, as reported previously, apathy is one of the most prevalent and persistent NPS among NH residents [7]. Some evidence suggests that apathy among residents with dementia could benefit from individualized treatment using non-pharmacologic interventions [16]. Accordingly, when developing an individual care plan including m-CGA the staff takes into account the residents’ past preference for activities, functional resources and environmental factors [49]. These areas were emphasised in the execution of CC-meetings in the present study and could be a plausible explanation for the reduction of apathy. Research has shown that reducing apathy might increase quality of life [50] and higher level of apathy is associated with carer distress [51]. Furthermore, CC may have increased the awareness of the residents’ psychosocial situation, accompanied with an adapted way of interacting with these residents. However, the present study did not record the specific initiated nursing interventions following the CC, neither observed if the care interactions became more health promoting.

This study implemented a complex intervention that was designed to facilitate the nursing home staff to improve the planning and coordination of the nursing interventions to meet residents’ needs and well-being possibly related to NPS. This was supported by a connected qualitative study in four of the included NH disclosing that nursing interventions and their evaluations developed to be more effective, timely, better planned and coordinated [32]. Thus, the residents’ needs, and problems were better handled after partaking in this trial.

### Strength and limitations

The strengths of our study are the cluster-randomised controlled design, the use of internationally recommended assessment tools, the large number of residents included, and the local anchoring with the NH RNs implementing the intervention. The real-world or pragmatic context for the study allowed nursing home staff to conduct their work without being observed and influenced, eliminating any potential occurrence of the Hawthorne or Observer effect. The NH did not receive additional resources, rendering work processes comparable to normal operation. In addition, it was adjusted for the effect of clustering.

Nevertheless, some limitations must be considered. It has a short follow up time. Special care units were excluded as they are not comparable to regular units regarding number of beds and staffing levels. The study was designed with limited blinding; a statistician did the analyses blinded for allocation to intervention and control groups. However, since this intervention involved the whole staff, blinding was not possible. Despite the large sample in our study, we did not achieve the number of NH (17 vs. 20) and residents (309 vs. 380) required by the power calculation. By randomizing the nursing homes and not the units within the nursing homes, differences between the nursing homes could potentially influence the results. However, by doing a stratified randomization on the size of the nursing homes, this limitation is considered to be small. The use of proxies (RN) to conduct the outcome assessments are always based on their knowledge and observations of the residents and may not fully be in accordance with the resident’s situation. On the other hand, reliability and validity of proxy data are found to be high for tasks of daily living and health conditions which may be easily observed [52]. In the Norwegian NH setting, most residents have cognitive impairment [3], and the proportion of residents with severe degree of dementia is considerable [3]. Thus, use of self-report questionnaires may not be trustworthy. We ensured that NPS was measured by a proxy who know the resident best [27]. Those assessing the residents knew the residents well and had got training/education in the assessment tools and the scoring, minimalizing the risk of unreliable assessment of residents. The baseline subsyndrome scores of NPI-Q in the present study were in line with another Norwegian study of NH residents [53]. However, the NPI-Q sum score and the subsyndrome scores were low and we cannot rule out the possibility of having a floor effect.

## Conclusions

The present cluster-randomised controlled trial found no change in total NPS after three months; however, the intervention group had less apathy at 3 months follow-up compared to those receiving care as usual. This finding should be further investigated in studies targeting one or a few NPS including only NH residents with dementia.

### Relevance for clinical practice

This study did not give conclusive findings for short-term effect of CC and m-CGA on NPS, and longer follow up is needed to suggest conclusions on clinical relevance. However, this type of interventions might have an impact on the quality of care by contributing to a systematic and regular assessment of NH residents’ individual needs and focusing on clinical decision-making. This could contribute to strengthening person-centered care by focusing on the resident’s health problems and needs. This study also demonstrates that implementing CC in combination with m-CGA is a feasible way to develop and follow-up individual care plans, as shown previous research [23]. Based on the experiences from this study, implementation strategies should take into account time for training of the staff and time for the nursing home as organisation to adapt to changes in routines. Hence, there is a need for more research on how nursing interventions are planned and implemented in NH. There is also a need for more research focusing on the other dimensions of quality of care, i.e. how do CC and m-CGA contribute to better care planning and quality care for the residents with NPS.

## Data Availability

The datasets generated and/or analyzed during the current study will be available from the corresponding author on reasonable request. The reason is that it is not possible to share all data unless it is de-identified.
